# Ecological transition and sustainable development: integrated statistical indicators to support public policies

**DOI:** 10.1038/s41598-022-23085-0

**Published:** 2022-11-02

**Authors:** Francesco Rotondo, Paola Perchinunno, Samuela L’Abbate, Lucia Mongelli

**Affiliations:** 1 Department of Civil and Building Engineering, and Architecture, Polytechnic University of Marche, 60131 Ancona, Italy; 2 Department of Economics, Management and Business Law, University of Bari A. Moro, 70124 Bari, Italy; 3 National Institute of Statistics (ISTAT), Bari, Italy

**Keywords:** Ecology, Environmental sciences, Environmental social sciences

## Abstract

The evolution of the concept of sustainability and the availability of new statistical information requires constant checks on the set of indicators so that they accurately perform the task of representing well-being in our society. The Sustainable Development Goals refer to various development domains relating to environmental, social, economic, and institutional issues that have been placed at the basis of the Missions envisaged by the National Recovery and Resilience Plan (NRRP). Specifically, the subject of ecological transition and the related statistical indicators and the evaluations of the effectiveness of the programming implemented by the NRRP to pursue it in practice are of significant interest. The numerous data available were analyzed at a regional level through multivariate statistical methodologies (Totally Fuzzy and Relative method) capable of synthesizing the various information to evaluate the territorial adequacy of the economic planning of its various components. Through the representation on a GIS basis of the geographical distribution of the synthesis values of the fuzzy indices, the paper highlights the different starting point existing between Italian regions. So, these integrated statistical indicators can help public policies to be oriented in a more coherent way with their environmental declared objectives. Starting from the availability of multiple data, it is developed an integrated approach to the evaluation of the local government policies in place and to monitor the progress of subsequent interventions by the Italian government.

## Introduction

The evolution of the concept of sustainability and the availability of new statistical information requires constant checks on the set of indicators so that they accurately carry out the task of representing well-being in our society. Statistics is called to contribute to progress towards sustainability by guaranteeing its own service, which consists in providing ever better evidence, to accompany every phase of the construction of sustainable development. The Sustainable Development Goals refer to different domains of development that go well with the Missions envisaged by the National Recovery and Resilience Plan (NRRP). Specifically of great interest is the issue of ecological transition and the statistical indicators connected to it.

The ecological transition is the process of technological innovation to achieve change in our society considering compliance with the criteria for environmental sustainability. At the heart of the United Nations 2030 Agenda is precisely the theme of sustainable development in the economic, social and environmental dimensions^1,2^. Among the many objectives are those concerning urgent ecological measures to combat climate change, to protect the oceans, seas and marine resources and to manage forests by combating desertification.

Here are some key points to ensure the ecological transition:

1. Renewable sources: without an increase in investments in forms of energy that respect the resources coming from the natural world and therefore do not pollute and do not run out, it will not even be possible to achieve the European objectives.
2. Electric mobility: use of electricity for transport. The transport sector is one of the main contributors to air pollution. To reach the goal of 6 million electric vehicles by 2030, we need to really invest in urban, regional and electric transport mobility.
3. Digital energy: use of advanced digital technologies along the energy supply chain.
4. Energy storage, i.e. energy storage and storage with a view to greater energy efficiency.
5. Smart building: creating buildings in which energy efficiency systems are managed in an intelligent and automated way.
6. Circular economy: an economy designed to be able to regenerate itself, where any waste is seen as resources and there is no waste. A new vision of production, consumption, disposal, and logistics.
7. Agroecological model: reduce the use of pesticides and provide for a further increase in the area to be dedicated to organic farming. It is necessary to intervene on the intensive farming system to reduce emissions and impacts on health and the environment.

The present work deals with analyzing the relationships between the domains of the Sustainable Development Goals (SDGs) and the Missions envisaged by the National Recovery and Resilience Plan (NRRP). Specifically, we refer to Mission 2 (Green Revolution and Ecological Transition) and therefore to the statistical indicators connected to it^3^.

The numerous available data have been analyzed at regional level through multivariate statistical methodologies (Totally Fuzzy and Relative method) capable of synthesizing the different information to evaluate the current situation in the different components (MC1, MC2, MC3, MC4).

Specifically, the strength of this research consists in the possibility of analyzing the different components of mission 2 (Green Revolution and Ecological Transition) of the NRRP through the construction of indicators deriving from the SDGs. These sets of indicators were then synthesized into a single fuzzy value that allows the overall situation of the different regions to be assessed and compared according to the different components.

In this way, we think we can translate in practice the principle described by UN Secretary-General António Guterres, that Sustainable Development Goals is Our Framework for COVID-19 Recovery and that we need to turn the recovery into a real opportunity to do things right for the future^4^.

The presence of multiple data updated to 2019 allows to develop an integrated approach to the evaluation of the government policies of the territory in place^5–7^ and to be able to monitor the progress of the subsequent intervention policies of the Italian government. It has already been affirmed by other authors^8^ the need to evaluate the effects of the NPPR on the other policies in progress in the European states in the period 2021–2027, demonstrating the importance of this evaluation for the successful outcome of the expenditure both from an economic point of view and under the environmental profile.

## The statistical indicators of the SDGS report (sustainable development goals)

### The project SDGs

The “Sustainable Development Goals” indicate what changes the nations and peoples of the world are committed to achieving, by virtue of a global consensus, obtained through a long, complex and difficult path of dialogue and international and interdisciplinary collaboration.

On 25 September 2015, 193 countries of the United Nations General Assembly adopted the 2030 Agenda for Sustainable Development, which sets out the global goals to end poverty, protect the planet and ensure prosperity to be achieved by 2030.

“*To continue in an economic and social development that ensures the satisfaction of the needs of the present generation without compromising the possibility of satisfying those of future generations*”^9^ this is the principle of equity between present and future generations and on the reduction of inequalities between countries and in countries.

Sustainable development is not just an environmental or just an economic or a social issue. The challenge that arises is precisely in the compatibility between economic growth, environmental protection, and social inclusion. The balance between the three areas of development, economic, social, and environmental, is therefore one of the key principles of Sustainable Development, together with the principles of *universality*, *integration and participation*.

Statistics is called to contribute to progress towards sustainability by guaranteeing its own service, which consists in providing ever better evidence, to accompany every phase of the construction of sustainable development.

Istat, like the other National Statistical Institutes, is called by the United Nations Statistical Commission to play an active role of national coordination in the production of indicators for the measurement of sustainable development and the monitoring of its objectives.

The 17 “Sustainable Development Goals (SDGs)” and the related 169 targets (sub-objectives) with which the three dimensions of sustainable development are declined, have extended the 2030 Agenda from the social pillar alone to the economic and environmental one, to these is added the institutional dimension, outlining a global action plan for the coming years.

Every year Istat publishes the Report on the SDGs^10^; the fourth report of August 2021 offers a first representation of the impact of the pandemic on the SDGs indicators. The fourth Report was last updated in February 2022.

The update summarizes:

- 367 statistical measures (of which 338 different).
- 135 indicators United Nations Inter Agency Expert Group (UN-IAEG), which constitute the global reference framework with the usual regional analysis, particularly useful for the observation of territorial imbalances.

It is a system of indicators of great complexity that sees within it both consolidated indicators available for most countries, and indicators that are not currently produced or that have not yet been exactly defined at the international level.

## Indicators of sustainability linked to the ecological transition

### The link between SDGs and NRRP

The Italian National Recovery and Resilience Plan (NRRP) is part of the Next Generation EU (NGEU) program, the 750-billion-euro package, consisting of about half of grants, agreed by the European Union in response to the pandemic crisis. The main component of the NGEU program is the Recovery and Resilience Facility (RRF), which has a duration of six years, from 2021 to 2026, and a total size of €672.5 billion (€312.5 billion grants, the remaining €360 billion loans at subsidized rates).

The Plan is developed around three strategic axes shared at European level: digitalization and innovation, ecological planning and social inclusion.

The missions of the NRRP are as follows:

- Mission 1: Digitalization, innovation, competitiveness, culture and tourism
- Mission 2: Green revolution and ecological transition
- Mission 3: Infrastructure for sustainable mobility
- Mission 4: Education and research
- Mission 5: Cohesion and inclusion
- Mission 6: Health.

**Figure 1:**
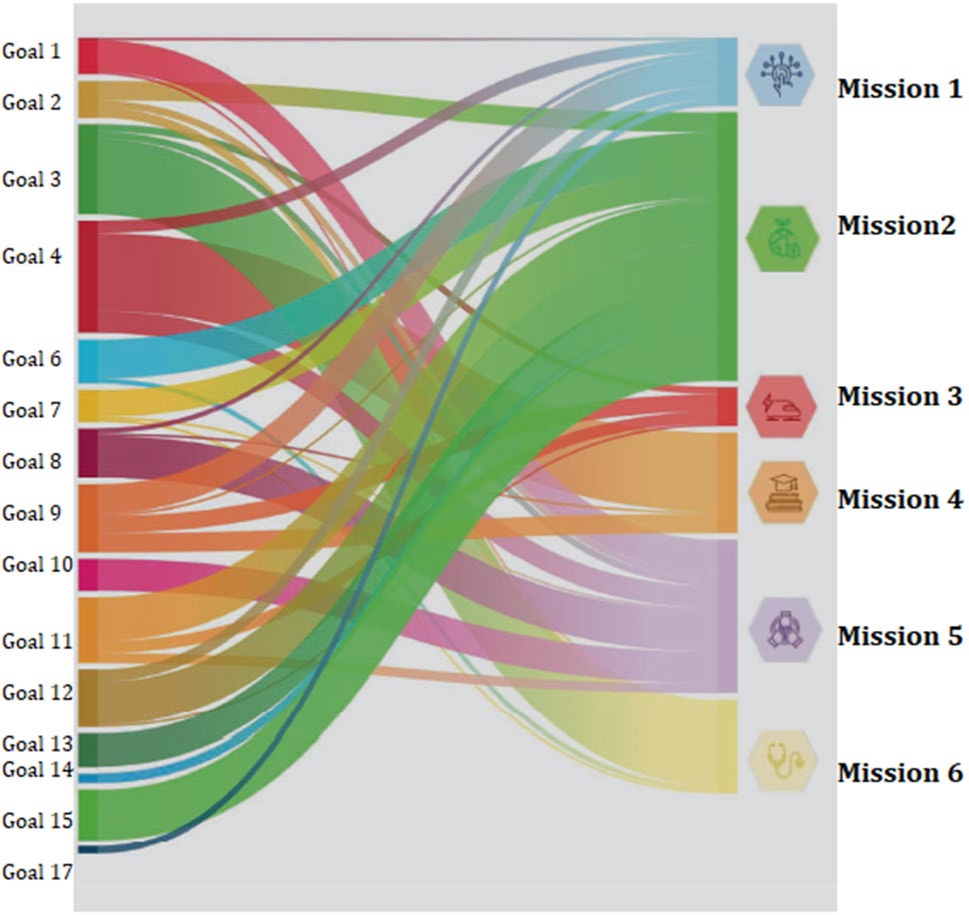
Relationships between SDGs indicators and NRRP missions.

With the aim of encouraging the debate on the use of sustainability indicators for monitoring the progress of the PNRR, a mapping of the correspondences between the 17 Sustainable Development Goals and the 6 Missions provided for by the NRRP is proposed (Fig. 1). In this way it is possible to identify the SDGs indicators that can be useful tools for achieving the missions of the NRRP.

Of particular interest for the purposes of our work is *Mission 2 (Green Revolution and Ecological Transition)* of NRRP*.* It provides for investments and reforms for the circular economy and to improve waste management, strengthen separate collection infrastructure and modernize or develop new waste treatment plants. Substantial tax incentives are provided to increase the energy efficiency of buildings, to achieve progressive decarbonization, to increase the use of renewable energy sources. In addition, the Mission devotes resources to enhancing the capacity of electricity grids, their reliability, security, and flexibility (Smart Grid) and water infrastructure. The Mission also includes the issues of territorial security, with prevention and restoration interventions in the face of significant hydrogeological risks, the protection of green areas and biodiversity, and those related to the elimination of water and soil pollution, and the availability of water resources.

The main components of this mission are:

- M2C1: Circular economy and sustainable agriculture
- M2C2: Renewable energy, hydrogen, grid, and sustainable mobility
- M2C3: Energy efficiency and upgrading of buildings
- M2C4: Protection of land and water resources.

The analysis of Mission 2 (Green Revolution and Ecological Transition) finds ample space in the SDGs creating important interconnections between the different indicators present in the individual Goals and the objectives of the Mission itself.

### The SDGs indicators to support the NRRP

The SDGs indicators selected for the analysis of Mission 2 (Green Revolution and Ecological Transition) of the NRRP, are descripted in Table 1. We considered 13 indicators, selected from Goals 2, 6, 7, 11, 12 and 15 which may be of significant interest for the achievement of Mission 2. These indicators will then be attributed to the individual components of the mission.

**Table 1:** Goal, indicators, measures e source of SDGs data.

Goal	Indicators	Measures	Source of data
Goal 2.4.1	Share of agricultural area allocated to sustainable and productive agriculture	Share of utilized agricultural area invested by organic crops	(Ministry of Agricultural, Food and Forestry Policies, 2019, percentage values)
Goal 2.4.1	Share of agricultural area allocated to sustainable and productive agriculture	Growth rate of organic crops	(Ministry of Agricultural, Food and Forestry Policies, 2019, percentage values)
Goal 6.1.1	Percentage of population benefiting from safely managed drinking water services	Irregularities in water distribution	(Istat, 2020, percentage values)
Goal 7.2.1	Share of energy from renewable sources in total final energy consumption	Electricity from renewable sources	(Terna Spa, 2019, percentage values)
Goal 7.2.1	Share of energy from renewable sources in total final energy consumption	Share of energy from renewable sources in gross final energy consumption	(GSE S.p.A.—Energy Services Manager, 2019, percentage values)
Goal 7.3.1	Energy intensity measured in terms of primary energy and GDP	Intensità energetica	(Enea, 2019, Tons of oil equivalent per million Euro)
Goal 7.3.1	Energy intensity measured in terms of primary energy and GDP	Energy intensity of the industrial sector	(Enea, 2019, Tons of oil equivalent per million Euro)
Goal 11.3.1	Relationship between land consumption rate and population growth rate	Waterproofing and soil consumption per capita	(Ispra, 2019, m^2^ per inhabitant)
Goal 11.6.1	Percentage of municipal solid waste regularly collected with an adequate final delivery of the total waste produced in the city	Landfilling of municipal waste	(Ispra, 2019, percentage values)
Goal 11.7.1	Average percentage of the urbanized area of cities that is used as a public space, by gender, age and people with disabilities	Impact of urban green areas on the urbanized surface of cities	(Istat, 2019, m^2^ per 100 m2 of urbanized area)
Goal 12.5.1	National recycling rate, tons of recycled material	Separate waste collection	(Istat elaboration on Ispra data, 2019, percentage values)
Goal 15.3.1	Share of degraded territory on the total land area	Soil sealing from artificial cover	(ISPRA, 2019, percentage values)
Goal 15.3.1	Share of degraded territory on the total land area	Fragmentation of natural and agricultural territory	(ISPRA, 2019, percentage values)

*Source*: our elaboration on SDGs.

The indicators were chosen based on their relevance to the objectives of the mission and on the availability of data on a regional basis. For each main component we can use the following indicators:

- M2C1: Circular economy and sustainable agriculture:
  - Share of utilized agricultural area invested by organic crops
  - Growth rate of organic crops
  - Delivery of municipal waste to landfill.
  - Separate waste collection
- M2C2: Renewable energy, hydrogen, grid and sustainable mobility:
  - Electricity from renewable sources
  - Share of energy from renewable sources in gross final energy consumption
- M2C3: Energy efficiency and upgrading of buildings
  - Energy intensity
  - Energy intensity of the industrial sector
- M2C4: Protection of land and water resources
  - Irregularities in water distribution
  - Sealing and soil consumption per capita
  - Soil sealing from artificial cover
  - Fragmentation of the natural and agricultural territory
  - Incidence of urban green areas on the urbanized surface of cities.

### The SDGs indicators at the level of territorial distribution in Italy

We carry out a first analysis by territorial distribution for the different sets of main components of Mission 2.

From a first analysis of the M2C1 indicators (Circular Economy and Sustainable Agriculture) it emerges that the *share of agricultural area destined for organic crops* is greater, especially in the Center and in the South of Italy. In 2019, the extent of organic farming in Italy reached 15.8% of the utilized agricultural area, almost double the EU average. However, the annual growth rate of the areas converted to organic farming or in the process of conversion (+ 1.8%) is the lowest since 2012 and is negative in the South, where for the second consecutive year there is a decrease (− 2.1% in the 2-year period 2017–2019). The dynamics of organic farming is an index of the spread of sustainable agricultural practices, which must be accompanied by measures that also consider the pressure on the environment generated by agriculture (Table 2).

**Table 2:** M2C1 indicators—Circular economy and sustainable agriculture by territorial distribution (year 2019).

Territorial distribution	Share of utilized agricultural area (UAA) under organic farming	Growth rate of organic crops	Landfilling of municipal waste	Separate collection of municipal waste
North	8.1	6.4	10.6	70.8
Center	21.0	4.3	29.3	59.2
South	19.7	− 0.4	31.2	53.6
**Italy**	**15.8**	**1.8**	**20.9**	**63.0**

*Source*: our elaboration on SDGs.

National values are in bold.

Also, in the Central and Southern Italy area there is the greatest *delivery of waste to landfills*. Waste cycle management is crucial for living conditions and global health. The share of municipal waste landfilled is steadily decreasing at national level. In 2019, in fact, the part sent to landfill is equal to 20.9% of the total, down compared to the previous year (21.5%). The *separate collection of municipal waste* represents a further important step in view of the objective of reducing the amount of waste returned to the environment and, more specifically, of the delivery of waste to landfills. The 18.5 million tons of differentiated RU in 2019 represent 61.3% of national production, a share almost doubled compared to ten years ago and up from last year by 3.1 percentage points. Despite the evident progress, Italy is still marked by a considerable delay compared to the regulatory objectives, having not yet reached, in 2019, the target of 65% of separate collection planned for 2012. Critical issues are also observed in relation to the substantial territorial gaps, which disadvantage the Center and the South compared to the North, despite the distances have been reduced in recent years.

Regarding the M2C2 Mission (Renewable Energy, Hydrogen, Network and Sustainable Mobility), national and international energy policies have been committed for years to the enhancement of renewable energy sources, with the aim of decarbonizing the economy and guaranteeing the commitments made in the field of climate change. In 2019, one year after the expiry of the objectives of the European Union's Climate-Energy Package, fourteen Member States, including Italy, exceeded the target assigned at national level. In Italy, the *overall share of energy from renewable sources in gross final consumption* (CFL) of energy, equal to 18.2% (Table 3), a percentage slightly lower than the average of the EU27 (19.7%), is placed for the sixth consecutive year above the 17% target set for our country. However, for Italy to achieve the ambitious programs defined by the 2020 National Integrated Energy and Climate Plan, which set a 30% target for renewables by 2030, a further boost to production from renewable sources is necessary. The resources introduced by the National Recovery and Resilience Plan (NRRP) to achieve the “green revolution and ecological transaction” include significant investments in the energy field, focusing, among other components, on a further strengthening of the Sources from Renewable energy (FER).

**Table 3:** M2C2 indicators—Renewable energy, hydrogen, network and sustainable mobility by territorial distribution (year 2019).

Territorial distribution	Electricity from renewable sources	Share of energy from renewable sources in gross final energy consumption
North	32.8	32.7
Center	27.7	17.8
South	44.5	29.65
**Italy**	**34.9**	**18.2**

*Source*: our elaboration on SDGs.

National values are in bold.

The M2C3 Mission (Energy Efficiency and Upgrading of Buildings) devotes resources to enhancing the capacity of electricity grids, their reliability, safety, and flexibility (Smart Grid). Consistent with the objectives of reducing energy consumption pursued by European policies, the Italian figure for 2019 confirms the process *of reducing Italian energy intensity*, which marks a further contraction of 1.3%, reaching an overall negative balance compared to the last decade of 11.8%, with an average annual rate of change of − 1.2% (Table 4). The reduction in energy intensity is largely attributable to the effect of the measures in favor of energy efficiency, which, between 2011 and 2019, resulted in energy savings of 12 Mtoe/year, equal to 77% of the 2020 target set by the National Action Plan for Energy Efficiency 2017. A further acceleration of energy efficiency is expected, in the coming years, because of the investment plan envisaged by the NRRP, also linked to the redevelopment of the public and private building stock. At the sectoral level, the reduction in energy intensity is driven by improvements in industry, which, despite the slight increase in the last year, in 2019, with 92 toes per million euros, shows a decrease compared to 2009 of 17%, with an average annual rate of change of − 1.8%.

**Table 4:** M2C3 indicators—Energy efficiency and requalification of buildings by territorial distribution (year 2019).

Territorial distribution	Energy intensity	Energy intensity of the industry sector
North	86	845
Center	84	77
South	124	277
**Italy**	**92**	**100**

*Source*: our elaboration on SDGs.

National values are in bold.

The M2C4 Mission (Protection of the territory and water resources) also includes the issues of territorial safety, with prevention and recovery interventions, the protection of green areas and those related to the elimination of water and soil pollution.

Italy is among the European countries of the Mediterranean area that use groundwater, springs and wells the most; these represent the most important resource of fresh water for drinking water use on the Italian territory (84.8% of the total withdrawn). The efficiency of municipal drinking water distribution networks has been steadily deteriorating since 2008 for more than half of the regions. The share of families who complain of irregularities in the water supply service in their home is stable (equal to 8.6% in 2019) with more accentuated values in the Center and South of Italy (Table 5).

**Table 5:** M2C4 indicators—Protection of land and water resources by territorial distribution (year 2019).

Territorial distribution	Irregularities in water distribution	Waterproofing and soil consumption per capita	Soil sealing from artificial cover	Fragmentation of natural and agricultural territory	Impact of urban green areas on the urbanized surface of cities
North	3.1	409	8.5	43.8	11.0
Center	9	389	6.7	47.6	7.8
South	16.6	426	5.9	43.3	5.5
**Italy**	**8.6**	**357**	**7.1**	**44.3**	**8.5**

*Source*: our elaboration on SDGs.

National values are in bold.

Land degradation, understood as loss of ecological functionality, is monitored through the dynamics of land consumption, which Italy has committed to zero by 2030 with the National Strategy for Sustainable Development (2017). The “consumed” soil is that occupied by urbanization and made impermeable by artificial roofing (*soil sealing*). Excessive fragmentation of open spaces, however, is also a factor of degradation, since the barriers made up of buildings and infrastructures interrupt the continuity of ecosystems, making even unoccupied but not large enough spaces ecologically inert and unproductive. Moreover, in a fragile territory such as Italy, land consumption is also a significant factor of hydrogeological risk and deterioration of the landscape. The index of sealing and land consumption per capita in 2019 increases for the fifth consecutive year, resulting in 357 m^2^ per inhabitant. The soil sealed by artificial covers is equal to 7.1% of the national territory (8.5% in the North, 6.7% in the Center, 5.9% in the South).

According to Ispra estimates, 44.3% of Italy’s natural and agricultural land has a high or very high degree of *fragmentation*. A joint representation of the variations in fragmentation and *soil sealing* over the last two years summarizes recent trends in land consumption and their impact on the environment and landscape.

A further objective for 2030 is to provide universal access to safe, inclusive, and accessible public green spaces, for women and children, the elderly, and people with disabilities. In 2019 the incidence of urban green areas on the urbanized surface of cities is equal to 8.5% in Italy with slightly higher values in the North and less elevated in the South.

## A multidimensional approach for the identification of the area that require NRRP interventions

### The Fuzzy approach

The development of fuzzy theory initially stems from the work of Zadeh^11^ and subsequently was conducted by Dubois and Prade^12^. The fuzzy theory develops starting from the assumption that each unit is not univocally associated with only one but simultaneously with all the categories identified based on links of different intensity (degrees of association).

The first measurement based on the fuzzy set theory, named TF (Totally Fuzzy), was suggested by Cerioli and Zani^13^. This logic can be applied to both continuous and ordinal variable cases. However, in the latter case, the maximum and minimum values can be determined by assuming the value of the lowest category as minimum and the highest as maximum.

Cheli and Lemmi^14^ have proposed a generalization of this approach, called *Totally Fuzzy and Relative* (TFR). This method is also called “totally relative” because the value of the membership function is entirely determined by the relative position of the individual in the distribution of the population. The fuzzy TFR approach consists in defining the measurement of an individual’s *degree of belonging* to the totality fuzzy, included in the interval between 0 (with an individual who does not demonstrate a clear belonging) and 1 (with an individual who demonstrates a clear belonging).

Given a set ***X*** of elements, any blurred subset $x \in {\rm X}$ ***A*** of ***X*** is defined as follows:

1 $${\mathbf{A}} = \left\{ {{\mathbf{X}},f_{A} (x)} \right\}$$

where $f_{A} (x):{\mathbf{X}} \to \left[ {0,1} \right]$ is called the blurred subset membership *function ****A*** and indicates the degree of belonging of *x* to ***A*** where:

- if $f_{A} (x) = 0$ it occurs that *x* does not belong to **A;**
- if $f_{A} (x) = 1$ it occurs that *x* belongs completely to ***A*;**
- if $0 < f_{A} (x) < 1$ it occurs that *x* partially belongs to A, with a degree of belonging that is greater the closer it is to 1.

If we suppose to observe k indicators for each family, the function of belonging of the i-th family to the blurred subset can be defined as follows:

2 $$f(x_{i} ) = \frac{{\sum\limits_{j = 1}^{k} {g(x_{ij} ).w_{j} } }}{{\sum\limits_{j = 1}^{k} {w_{j} } }}\begin{array}{*{20}c} , & {i = 1, \ldots ,n} \\ \end{array}$$

where g(x_ij_) measures the probability of membership of each unit and w_j_ derives from a weighting system, as given by generalizing the one proposed by Cerioli and Zani^13^:

3 $$w_{j} = \ln \left[ {1/\overline{{g(x_{j} )}} } \right],\quad \quad \quad where\quad \overline{{g(x_{j} )}} = \frac{{\sum\limits_{i = 1}^{n} {g(x_{ij} )} }}{n}\quad \quad (j = 1,2,...,k).$$

When the average function $\overline{g({x}_{j})}=1$ the corresponding weight wj is equal to zero, while when $\overline{g({x}_{j})}=0$ w_j_ is not defined, or rather X_j_ is not an appropriate indicator for that collective.

To avoid possible problems due to the unbalanced frequency distribution of indicators, that is, high frequencies associated to single modalities or to extreme values, alternative specification of g(x_ij_) may be used, as proposed by Cheli and Lemmi^14^:

4 $$g(x_{ij} ) = \left\{ {\begin{array}{*{20}l} 0 \hfill & {{\text{ if}}\quad x_{ij} = x_{j}^{(1)} } \hfill \\ {g(x_{j}^{(k - 1)} ) + \frac{{H(x_{j}^{(k)} ) - H(x_{j}^{(k - 1)} )}}{{1 - H(x_{j}^{(1)} )}}} \hfill & {{\text{ if}}\quad x_{ij} = x_{j}^{(k)} ;\quad k > 1} \hfill \\ \end{array} } \right.$$

where *x*_*j*_^*(1)*^*,* ….. *x*_*j*_^*(m)*^ represent the values of the variable *X*_*j*_ , ordered according to an increasing *risk of membership* (so *x*_*j*_^*(1)*^ and *x*_*j*_^*(m)*^ denote, respectively, the minimum and the maximum risk), and *H(x*_*j*_^*(k)*^*)* is the breakdown function of the ordered variable *X*_*j*_^*(k)*^.

The indices were chosen to identify different levels, related to the aspects of Mission 2 (Green Revolution and Ecological Transition). The indices were grouped into several sets characterized by different situations in the different components considered for the mission (M2C1: Circular economy and sustainable agriculture, M2C2: Renewable energy, hydrogen, grid, and sustainable mobility, M2C3: Energy efficiency and redevelopment of buildings and M2C4: Protection of the territory and of the water resource). The Total Fuzzy and Relative method was applied to the data of all the Italian regions, obtaining a value of the individual weights w_i_, which varies according to the level of importance in determining the quality of the situation.

### The results of the application of Totally Fuzzy and Relative approach

Once the sets of indicators for the 3 components considered (M2C1, M2C2-3, M2C4) were identified, the minimum, maximum and average values for each indicator of the different sets were detected. For each set of indicators, the fuzzy value and its values attached to it have been calculated. Of particular interest is the analysis of *w*_*i*_* weights* that indicate the relevance of the indicator on the considered set. As already specified, high values of this indicator denote a strongly discriminating condition in the determination of the result (Table 6).

**Table 6:** Results of the application of the TFR method in relation to the distribution function and the weights of the different indices.

Set of Indicators	Indicators	Minimum	Maximum	Mean	Gmean	Weight *w*_*j*_
M2C1: Circular economy and sustainable agriculture	Share of utilized agricultural area invested by organic crops	5.3	36.4	13.6	0.1	1.2
	Growth rate of organic crops	− 22.5	31.3	3.8	0.7	0.3
	Delivery of municipal waste to landfill	1.3	90.0	27.5	0.6	0.5
	Separate waste collection	38.5	77.5	61.7	0.6	0.5
M2C2: Renewable energy, hydrogen, network and sustainable mobilityM2C3: Energy efficiency and upgrading of buildings	Electricity from renewable sources	8.6	264	66	0.2	1.5
	Share of energy from renewable sources in gross final energy consumption	8.8	92.4	29.9	0.3	1.3
	Energy intensity	62.2	177	97.6	0.6	0.5
	Energy intensity of the industrial sector	51.7	450	131	0.8	0.3
M2C4: Protection of land and water resources	Irregularities in water distribution	1.4	31.2	8.5	0.7	0.4
	Sealing and soil consumption per capita	241	568	412	0.5	0.6
	Soil sealing from artificial cover	2.1	12.1	6.3	0.6	0.6
	Fragmentation of the natural and agricultural territory	1.2	66.7	39.2	0.4	0.8
	Incidence of urban green areas on the urbanized surface of cities	3.3	12.7	8.0	0.5	0.7

*Source*: our elaboration on SDGs.

In our case the values of the weights of the indicators relating to the share of utilized agricultural area invested by organic crops (*w*_*i*_ = 1.2), and those relating to electricity from renewable sources (*w*_*i*_ = 1.5) and the share of energy from renewable sources on gross final energy consumption (*wi* = 1.3) are particularly discriminating. Conversely, the weights of the indicators relating to the growth rate of organic crops and the energy intensity of the industrial sector (both equal to *w*_*i*_ = 0.3) appear less discriminating.

As a result of the application, we have classified the Italian regions based on fuzzy values obtaining the classification illustrated in Table 7. Recall that high values are significant of regional situations in good health and therefore do not require national interventions, vice versa low values are significant of situations of difficulty or lack of the presence of certain goods.

**Table 7:** Composition in absolute values and percentages of regions by membership of fuzzy classes.

Fuzzy value	Number of regions			%		
	MC1	MC2–MC3	MC4	MC1	MC2-MC3	MC4
0,0 _┤_0,2	2	9	0	9%	41%	0%
0,2 _┤_0,4	3	6	5	14%	27%	23%
0,4 _┤_0,6	10	2	11	45%	9%	50%
0,6 _┤_0,8	7	3	4	32%	14%	18%
0,8 _┤_1,0	0	2	2	0%	9%	9%
**Total**	**22**	**22**	**22**	**100**	**100**	**100**

Total values are in bold.

### Spatial distribution of fuzzy values

In this paper, the representation of the values attributed to the individual geographic areas, corresponding to the regions, occurs through cartograms, associated with “natural” interval classes, defined within the distribution.

**Figure 2:**
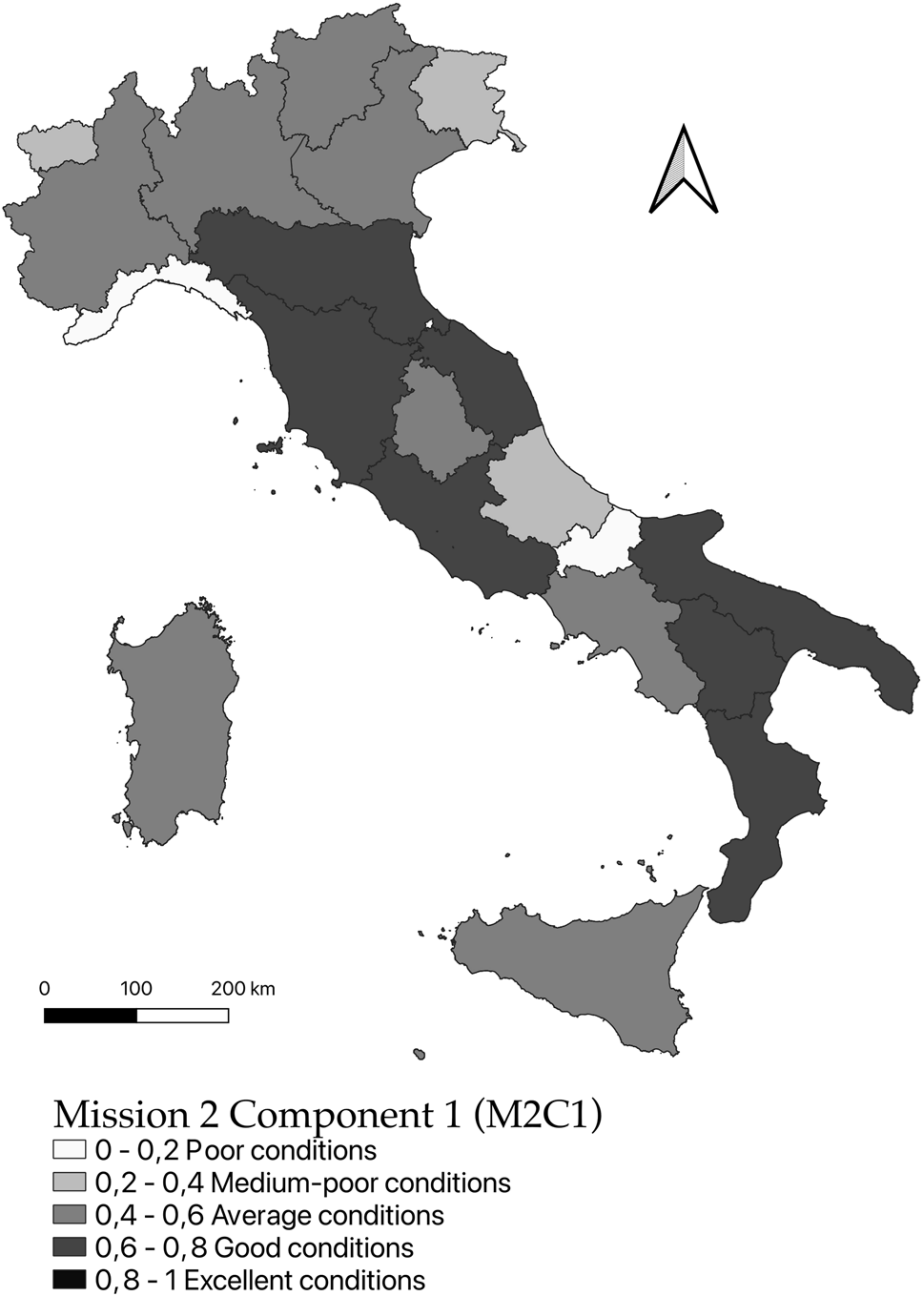
Territorial distribution by region of the fuzzy values for Mission 2 Component 1 (Circular Economy and Sustainable Agriculture).

Applying the *Total Fuzzy and Relative* method on the data of all Italian regions, as described in the previous paragraph, three synthetic indices were obtained that describe the territorial distribution of the indicators that are represented in the Fig. 2. It thus emerges that for the set related to the indicators related to the M2C1 mission (Circular Economy and Sustainable Agriculture) most of the regions are in an average condition while 5 regions have strong shortcomings (Liguria, Molise, Valle d’Aosta, Friuli-Venezia Giulia, and Abruzzo). The Fig. 2 clearly shows that, in this field, as in others following, NRRP can’t be applied in the same way because, once again, it risks exalting regional inequalities rather than shortening them, contributing to the failure of cohesion policies conducted with other funds.

It’s a very new subject (also because National states are in an exceptional hurry to spend this money), but European Committee of the Regions seem already knowing examples of synergies between the Sustainable Development Goals and the national recovery and resilience plans^15,16^.

**Figure 3:**
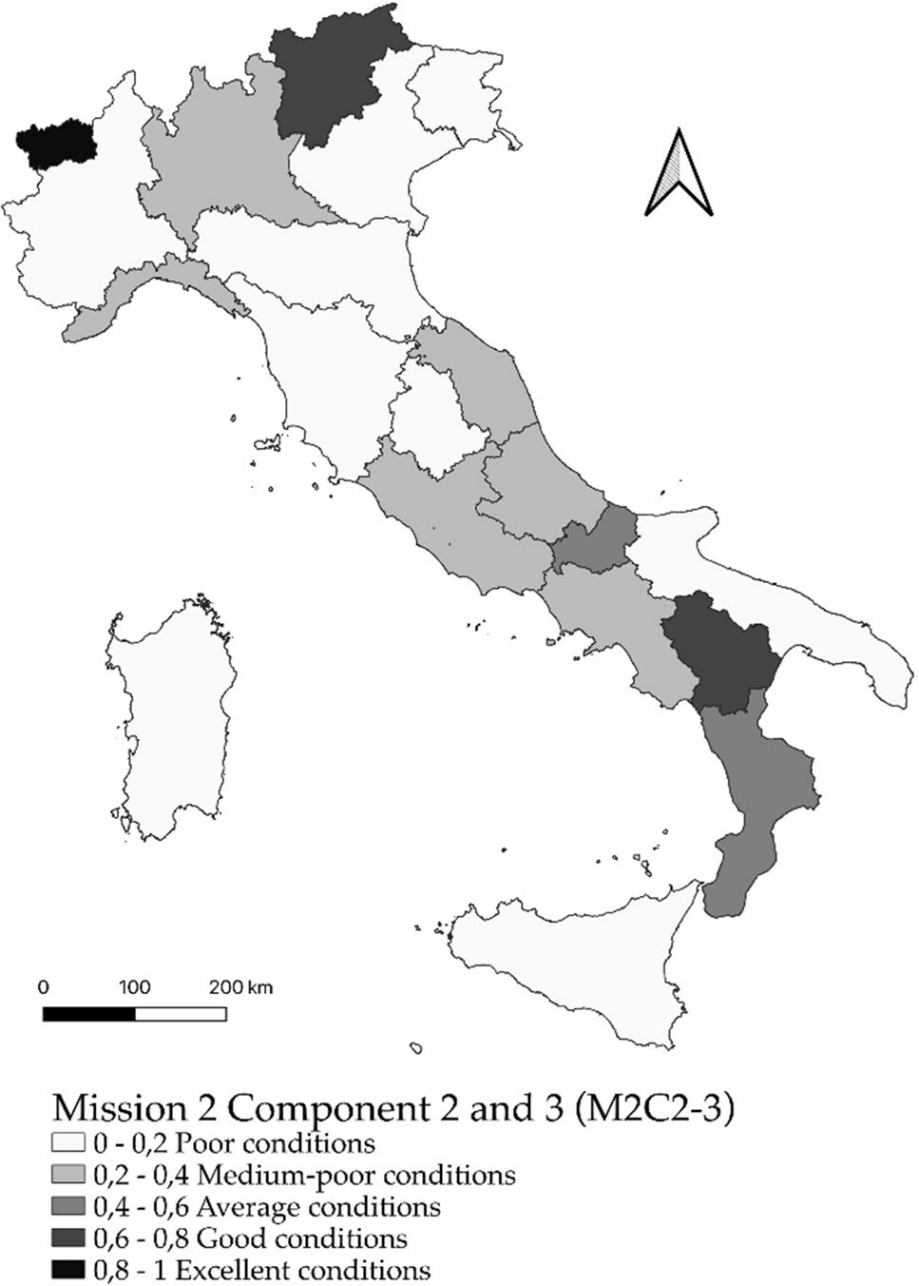
Territorial distribution by region of the fuzzy values for Mission 2 Components 2 (Renewable energy, hydrogen, network and sustainable mobility) and Component 3 (Energy efficiency and upgrading of buildings).

Regarding the missions M2C2-3 (Renewable energy, hydrogen, network and sustainable mobility; Energy efficiency and redevelopment of buildings) there is instead a greater equidistribution in all classes (Fig. 3) with the presence of 9 regions in conditions of need (Sicily, Puglia, Friuli-Venezia Giulia, Umbria, Tuscany, Piedmont, Veneto, Emilia Romagna and Sardinia).

**Figure 4:**
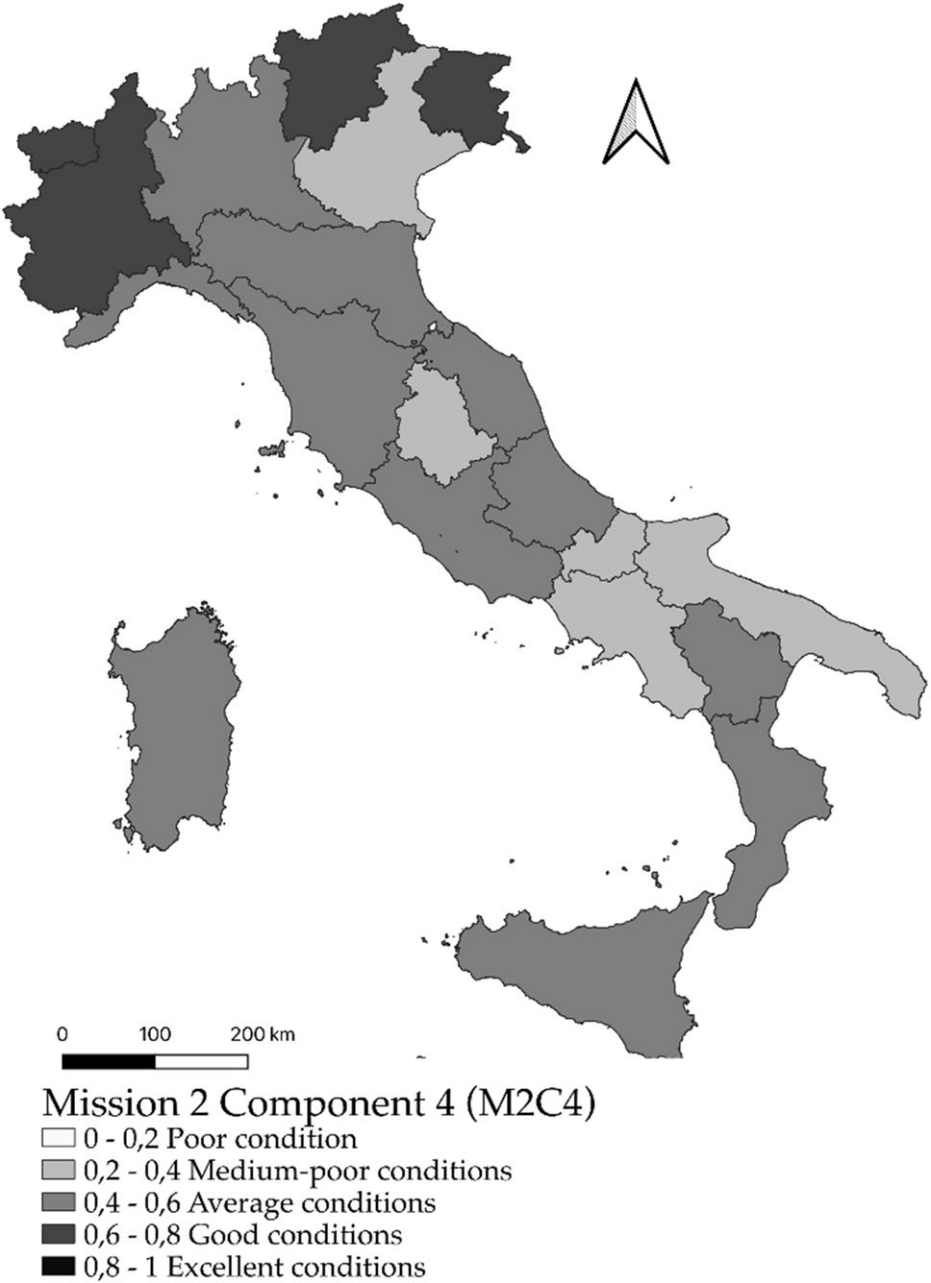
Territorial distribution by region of the fuzzy values for Mission 2 Component 4 (Protection of land and water resources).

Finally, for the M2C4 mission (Protection of land and water resources) the situation appears like MC1 (Fig. 4) with 11 regions on average and 5 regions with shortage situations (Puglia, Molise, Veneto, Umbria and Campania).

## Conclusions

The national policy decisions to be taken in the decade 2020–2030 and to be put into practice are, at the same time, simple and very complex but crucial for the relationship between man and nature in realizing a real sustainable development, overcoming the geological era defined the Anthropocene, by the Nobel prize winner Paul Kruzten^17^. By providing summary data, sustainability indicators can guide the analysis and effectively orient political, economic, and social decision-making processes in the regional government, that is the sub-national dimension of European policies^18^.

The Next Generation EU (NGEU) promotes development based on sustainability and represents the highest investment that Europe has ever put in place. Italy, Spain, Greece, and Portugal, in that order, are to be allocated over 44% of the NGEU total for the 27 countries. It is evident, from an economic point of view, the importance of spending the available funds not only in the prescribed time frame (2026), but also and above all in the most capable ways of multiplying the effects of economic resources to make them increasingly capable of being lasting investments.

Therefore, the territorial disparities that this work has highlighted between the Italian regions, by evaluating mission 2 of the NRRP and its components through the sustainable development goals of the 2030 Agenda, must become elements capable of guiding the methods of resource distribution. Investments must not be distributed in the same way to all Italian regions because they do not start from the same conditions^19^.

The competitive criterion that is being used in the calls already issued for the implementation of the NRRP risks increasing these disparities because it is likely that regions that are more economically equipped and with greater capacity to respond to competitive calls (because, for example, equipped with a larger, more competent, regional bureaucracy) can draw more resources.

The results of the fuzzy analysis developed in this work show that it is necessary to integrate the usual methods of distributing funds with adequate tools for territorial rebalancing that in each subject can support precisely the regions that start from the most disadvantaged situations. From the analyzes carried out, among other things, it emerges that not in all cases the territorial disparities reproduce the classic gap between North and South, but the disparities are also distributed within the two classic Italian macro-areas.

Unfortunately, Italy has experienced a long history of asymmetrical development and territorial imbalances that have never been compensated precisely because the resources destined to compensate for these disparities and promote cohesion, as already noted in some in-depth analyzes^20^, have end continued to strengthen the stronger areas.

In conclusion, we are now aware that the conditions for rebalancing different starting situations do not arise spontaneously, especially in the weaker areas, but require careful public policies that contribute to creating them. Taking note, also from the results of this study, of the different starting situations of the Italian regions with respect to the sustainability objectives, it is necessary to commit to distribute the huge resources of the NRRP also considering these imbalances. Pursuing sustainable development is too important an objective to leave its implementation to chance.

## Data Availability

The datasets used and analyzed during this study are available from the corresponding author upon reasonable request.
